# Reduced protein synthesis in schizophrenia patient-derived olfactory cells

**DOI:** 10.1038/tp.2015.119

**Published:** 2015-10-20

**Authors:** J A English, Y Fan, M Föcking, L M Lopez, M Hryniewiecka, K Wynne, P Dicker, N Matigian, G Cagney, A Mackay-Sim, D R Cotter

**Affiliations:** 1 Department of Psychiatry, University College of Dublin School of Biomolecular & Biomedical Science, Royal College of Surgeons in Ireland, Dublin, Ireland; 2 Eskitis Institute for Drug Discovery, Griffith University, Brisbane, QLD, Australia; 3 University College of Dublin School of Biomolecular & Biomedical Science, Dublin, Ireland

## Abstract

Human olfactory neurosphere-derived (ONS) cells have the potential to provide novel insights into the cellular pathology of schizophrenia. We used discovery-based proteomics and targeted functional analyses to reveal reductions in 17 ribosomal proteins, with an 18% decrease in the total ribosomal signal intensity in schizophrenia-patient-derived ONS cells. We quantified the rates of global protein synthesis *in vitro* and found a significant reduction in the rate of protein synthesis in schizophrenia patient-derived ONS cells compared with control-derived cells. Protein synthesis rates in fibroblast cell lines from the same patients did not differ, suggesting cell type-specific effects. Pathway analysis of dysregulated proteomic and transcriptomic data sets from these ONS cells converged to highlight perturbation of the eIF2α, eIF4 and mammalian target of rapamycin (mTOR) translational control pathways, and these pathways were also implicated in an independent induced pluripotent stem cell-derived neural stem model, and cohort, of schizophrenia patients. Analysis in schizophrenia genome-wide association data from the Psychiatric Genetics Consortium specifically implicated eIF2α regulatory kinase EIF2AK2, and confirmed the importance of the eIF2α, eIF4 and mTOR translational control pathways at the level of the genome. Thus, we integrated data from proteomic, transcriptomic, and functional assays from schizophrenia patient-derived ONS cells with genomics data to implicate dysregulated protein synthesis for the first time in schizophrenia.

## Introduction

Schizophrenia is among the most disabling of human diseases, with poorly understood pathophysiology.^1^ Many cellular and molecular phenomena have been described in neurons of schizophrenic patients, mostly based on post-mortem, neuroimaging and pharmacological data; however, there is still no clear understanding of the cellular and molecular mechanisms underlying the disease. One of the major challenges has been accessing appropriate cells, tissues and animal models that are relevant to the disease pathology. We reasoned that protein expression changes in olfactory neurosphere-derived (ONS) cells may provide novel insights into cellular processes that are dysregulated in schizophrenia.

Patient-derived neural cell models of schizophrenia such as those derived from nasal biopsy of the olfactory mucosa, used here, do not require genetic reprogramming and can be obtained from adults with complex genetic disorders.^2, 3^ Research in schizophrenia patient-derived olfactory cells has already revealed insights into specific microRNA effects that are in keeping with the molecular changes associated with schizophrenia,^4, 5, 6^ as well as disease-associated alterations of cell cycle, cellular adhesion and migration.^7, 8^ Disease-associated alterations in migration, along with dysregulated cytoskeletal genes and proteins, were also observed in neural progenitor cells generated from schizophrenia-derived induced pluripotent stem cells (iPSCs).^9^ Our objective was to identify disease-associated cellular processes in schizophrenia patient-derived ONS cells. Our strategy was to use discovery-based protein expression profiling to identify significantly altered processes and pathways and examine those altered pathways at a functional level.

## Materials and methods

For more detailed information, please refer to extended experimental procedures in Supplementary Information.

### Human ONS cells

To identify dysregulated cellular pathways, olfactory mucosa biopsies were obtained from schizophrenia patients (*n*=9) and healthy age- and sex-matched control (*n*=9) patients (Supplementary Table 1). Olfactory biopsies were dissociated and cultured as neurospheres in epidermal growth factor and basic fibroblast growth factor, after which the patient- and control-derived ONS cell lines were grown under standard conditions, with all experiments performed at passages 5–11, as previously described.^8, 10^

### Mass spectrometry

For proteomic analysis of the ONS cells using label-free liquid chromatography-mass spectrometry (LC-MS),^11^ 1 μg of protein from each ONS cell line (*n*=18) was run in triplicate on a Thermo Scientific (World wide vendor http://www.thermoscientific.com) LTQ ORBITRAP XL mass spectrometer. Label-free quantification was performed using Max Quant (V1.3.0.2; www.maxquant.org). Raw mass spectrometry (MS) files used in this experiment have been uploaded to the proteomics data repository PRIDE to facilitate data dissemination. For targeted MS of ribosomal proteins, schizophrenia or control samples were pooled and injected in triplicate on a Thermo Q-Exactive. Peptides were targeted using a mass selective MS1 single-ion monitoring scan at a resolution of 35 000 (for peptide isolation list see Supplementary Dataset 1). The Skyline software package (v2.1.0.4936; https://skyline.gs.washington.edu) was used for MS1 filtering^12^ and retention time prediction^13^ of target peptides (Supplementary Figure 1a). Western blotting for elF2α, RPL13A and RPL18A was used to confirm the above changes in schizophrenia and control ONS cells (Supplementary Figure 1b). Please refer to online methods for further details.

### Protein translation assay in ONS cells and fibroblasts

Global protein synthesis was quantified in schizophrenia- and control-derived ONS cells, and fibroblasts from the same individuals. The methionine analog, L-homopropargylglycine (HPG), was incorporated into newly synthesized proteins in culture and was labeled with a fluorescent tag using copper-catalyzed azide–alkyne cycloaddition, according to the manufacturer's protocol (‘Click-IT', Invitrogen, Carlsbad, CA, USA, Supplementary Methods). Experiments were performed on all nine schizophrenia patient-derived and nine control-derived ONS cell lines, at three HPG concentrations (25, 50 or 100 μM), at four time points (1, 2, 4 or 8 h). Duplicate plates were used for each time point. Differences in protein synthesis between schizophrenia- and control-derived cells were tested in a three-way analysis of variance (ANOVA) with disease status, HPG concentration and exposure time as main effects. Cell area was quantified with automated imaging and analysis using a fluorescent cytoplasmic stain (CellMask, Invitrogen).

### Statistical and bioinformatics analysis

**Figure 1:**
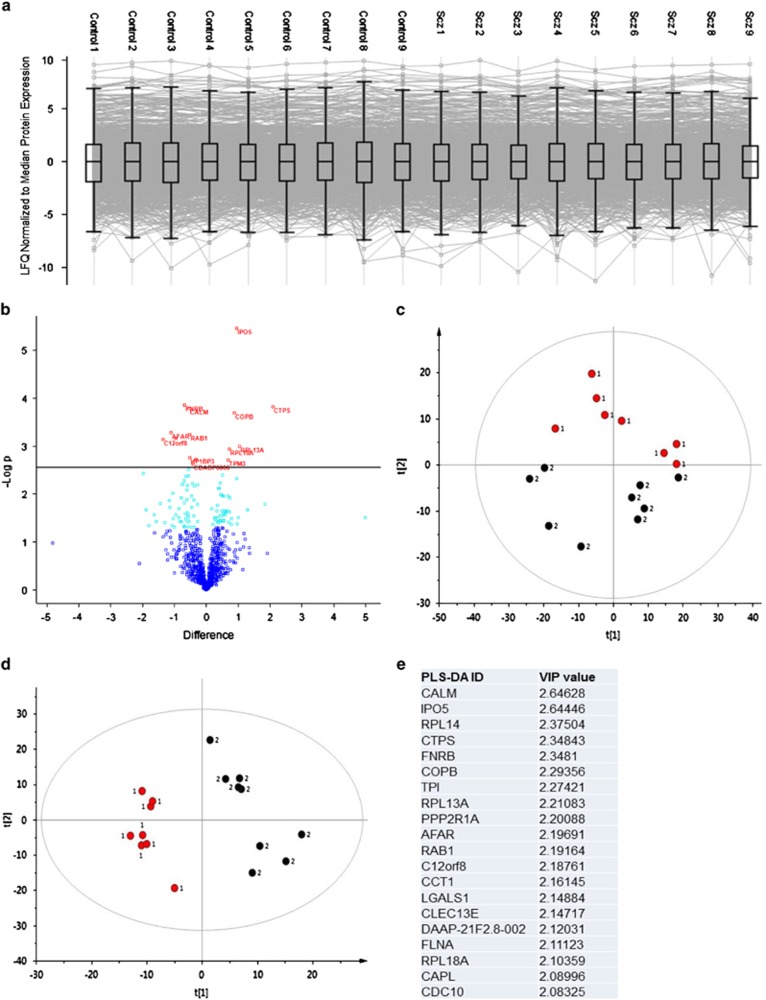
Protein expression profiling reveals discriminatory proteins between schizophrenia and control cell lines. (**a**) Line plots showing relative expression of the 859 protein profiled using label-free mass spectrometry (MS) across the 18 olfactory neurosphere-derived (ONS) cell lines, with overlaying box plots to illustrate the distribution and median of protein expression for each sample. (**b**) Volcano diagram plotting *t*-test difference versus significance (−log *P*-value) for all proteins. Differentially expressed proteins (*P*<0.05) are shown in turquoise and proteins significant following False Discovery Rate (FDR) correction are shown in red (see Supplementary Data set 2 for list). (**c**) Principal component analysis for differentially expressed proteins between the control (red circle) and schizophrenia (black circles) cell lines, *R*^2^=0.45 for a three-component model. (**d**) Partial least squares discriminant analysis (PLS-DA) score plot for control (red circle) and schizophrenia (black circles) cell lines, with an *R*^2^=0.4 and *Q*^2^=0.61 for a three-component model. (**e**) PLS-DA table of the variables of importance (VIP), which were deemed as having the best discriminatory power of the 102 significant proteins tested (Supplementary Data set 2) for the control and schizophrenia diagnostic groups. These VIPs are ranked in order, whereby the protein that exhibits the strongest discriminatory power has the highest numerical value among the 20 descriptor variables.

Statistical analysis of label-free LC-MS/MS data was performed in Perseus (V1.3.0.4; www.maxquant.org) and SIMCA-P+ (Version 13.0; Umetrics, Crewe, UK), as detailed in extended Supplementary Methods. Briefly, to identify significant expression changes at the protein level, a Student's *t*-test was applied at a 5% threshold, followed by a permutation-based false discovery rate (FDR) at a 10% threshold (Figure 1). As the objective of our study was to identify coordinated changes in pathways and cellular processes at the level of the proteome, we performed clustering analysis and pathway analysis on the full list of proteins identified as differentially expressed proteins by Student's *t*-test (*P*<0.05). Principle component analysis and Volcano plots were generated and performed in Perseus (V1.3.0.4), and Partial Least Squares-Discriminant Analysis (PLS-DA) was performed in SIMCA-P+ (Version 13.0; Umetrics). For investigations of potentially confounding effects of cigarette smoking and antipsychotic drug exposure on protein expression, please refer to online methods. Ingenuity pathway analysis was performed on statistically significant proteins and mRNA transcripts as per supplier's instructions (http://www.ingenuity.com/), detailed in Supplementary Methods.

### Testing for associations with schizophrenia in genome-wide association data

Single gene-based analysis was tested on the differentially expressed mRNA transcripts and proteins in the eIF2 signaling pathway (Table 1). Genome-wide association results were available from the Psychiatric Genetic Consortium.^14^ A gene-based test of the eIF2 signaling pathway was performed using the VEGAS software (http://gump.qimr.edu.au/VEGAS/), and a second analysis of gene sets of EIF2 signaling (*n*=48 genes), mammalian target of rapamycin (mTOR) signaling (*n*=40 genes) and EIF4 signaling (*n*=33 genes) was performed for enrichment with schizophrenia. The enrichment of the gene sets was tested using a competitive test of enrichment, GSEA v2.0 (ref. 15) as detailed in Supplementary Methods.

## Results

### Proteomic profiling reveals reduced expression of ribosomal proteins in schizophrenia

We measured the relative expression levels of 859 proteins identified across the 18 ONS samples, using label-free quantitative MS (Supplementary Data set 2). Whereas global protein expression was consistent across all samples (Figure 1a), we found 102 proteins to be differentially expressed between groups, 56 showing increased and 46 decreased expression in patient-derived cells (Figure 1b; Data set 2). Fifteen proteins remained significantly differentially expressed following permutation-based FDR, nine showing increased and six decreased expression. To identify co-regulated proteins and disease-associated pathways, we used the results from the low-stringency analysis (102 significant proteins @ *P*<0.05; Data set 2) for our clustering and pathway analysis. Principle component analysis of the differentially expressed proteins revealed a clear separation between schizophrenia and control diagnostic groups (Figure 1c). We then proceeded with partial least squares discriminant analysis of the differentially expressed proteins to provide insight into the causes of discrimination between the diagnostic groups, and to pull out the proteins that were variables of importance (Figure 1d). Analysis of the variable importance plots from the partial least squares discriminant analysis revealed the most powerful discriminatory proteins between groups (Figure 1e), including eIF2/4 pathway proteins (RPL14, RPL13A, RPL18A, PPP2R1A and FNRB), cytoskeletal proteins (FNRB, FLNA, C12orf8 and CDC10), mitochondrial proteins (TPI, LGALS1) and calcium-binding proteins (CALM and CAPL). Variable importance plots are ranked in order, where the protein that exhibits the strongest discriminatory power has the highest numerical value among the 20 descriptor variables (Figure 1e).

**Figure 2:**
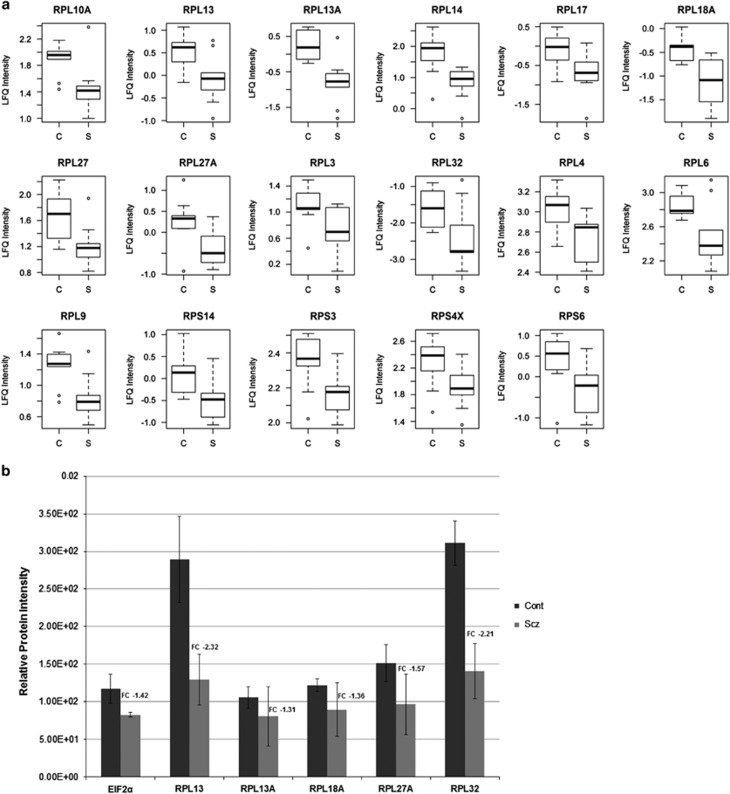
Reduced ribosomal protein expression in schizophrenia-derived olfactory neurosphere-derived (ONS) cells. (**a**) Boxplots representing the protein expression data from the discovery mass spectrometry (MS) analysis, which implicated 17 ribosomal proteins that were significantly decreased in expression in schizophrenia (S) patient-derived ONS cells compared with controls (C; *P*<0.05). The bottom and top of the boxes in each plot are the first (Q1) and third (Q3) quartiles, and the solid black band represents the median value or second quartile (Q2) for each RPL protein. The length of the rectangle from top to bottom is the interquartile range (IQR). The bottom whisker denotes either the minimum value or the first quartile minus 1.5 times the IQR (Q1–1.5*IQR), whichever is larger. The top whisker denotes the maximum value or the third quartile plus 1.5 times the IQR (Q3+1.5*IQR), whichever is smaller. (**b**) We used targeted LC-MS analysis to isolate and quantify peptides of interest, in which we confirmed a relative decrease in expression of eIF2α, RPL13, RPL13A, RPL18A, RPL27A and RPL32 proteins in pooled schizophrenia (Scz) ONS samples compared with pooled controls (Cont), which were run in triplicate. The relative intensity for each protein is represented on the y axis and error bars denote ±s.d. for each protein.

Notably, the uniform decreased expression of 17 ribosomal proteins (*P*<0.05; RPL4, RPL27A, RPL3, RPL27, RPL17, RPS6, RPL14, RPL9, RPL10A, RPL6, RPS4X, RPL18A, RPL32, RPS3, RPL13A, RPL13 and RPS14) was striking (Figure 2a). Analysis of the total MS signal intensity for all 67 cytoplasmic ribosome proteins identified found an 18% decrease in expression in the schizophrenia group (*P*=0.0004, likelihood-ratio test). In order to further confirm changes, we used an independent targeted MS approach^12^ to filter out target peptides of interest for relative quantification between groups. As the eIF2α protein is known to regulate the initiation of protein synthesis, and was significantly implicated at the mRNA level in the same cells,^10^ we included it on our target list of proteins for extended validation. Single-ion monitoring^12^ was used to isolate and quantify target peptides of interest, and relative quantification between the pooled schizophrenia and control groups was performed. As it is not statistically valid to apply a Student's *t*-test to pooled data, the relative fold change between the diagnostic groups was used to confirm the direction of change of target proteins. This analysis demonstrated that the levels of eIF2α, RPL13, RPL13A, RPL18A, RPL27A and RPL32 were reduced in schizophrenia patient-derived cells in comparison with controls (Figure 2b). Significant reductions in eIFα, RPL13A and RPL18A were also confirmed using western blot analysis on individual samples (see Supplementary Figure 1).

### Global protein synthesis is reduced in schizophrenia ONS cells

**Figure 3:**
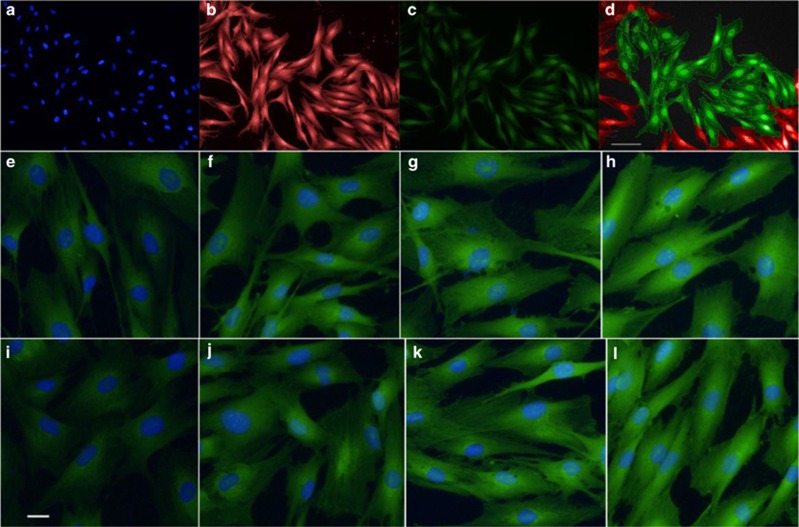
Quantification of global protein synthesis rates in schizophrenia patient-derived olfactory neurosphere (ONS)-derived cells. Automated quantification of global protein synthesis rates in ONS cell lines, and the same field of view for the nucleus ((**a**) 4′,6-diamidino-2-phenylindole (DAPI) stain), the cytoplasm ((**b**) CellMask stain), the L-homopropargylglycine (HPG) fluorescence ((**c**) azide fluorophore). (**d**) HPG-positive cells automatedly selected (green) and those discarded from the analysis because they overlapped the edge of the field (red). Control-derived ONS cells (**e**–**h**) and patient-derived ONS cells (**i**–**l**) were exposed for 1, 2, 4 and 8 h to HPG at three concentrations (25, 50 or 100 μM), with the 100 mM HPG concentration illustrated here. The scale bars are 100 μm (**a**–**d**) and 20 μm (**e**–**l**). Global protein synthesis rates were measured in all 18 cell lines, for three HPG concentrations, for at least 8 h.

**Figure 4:**
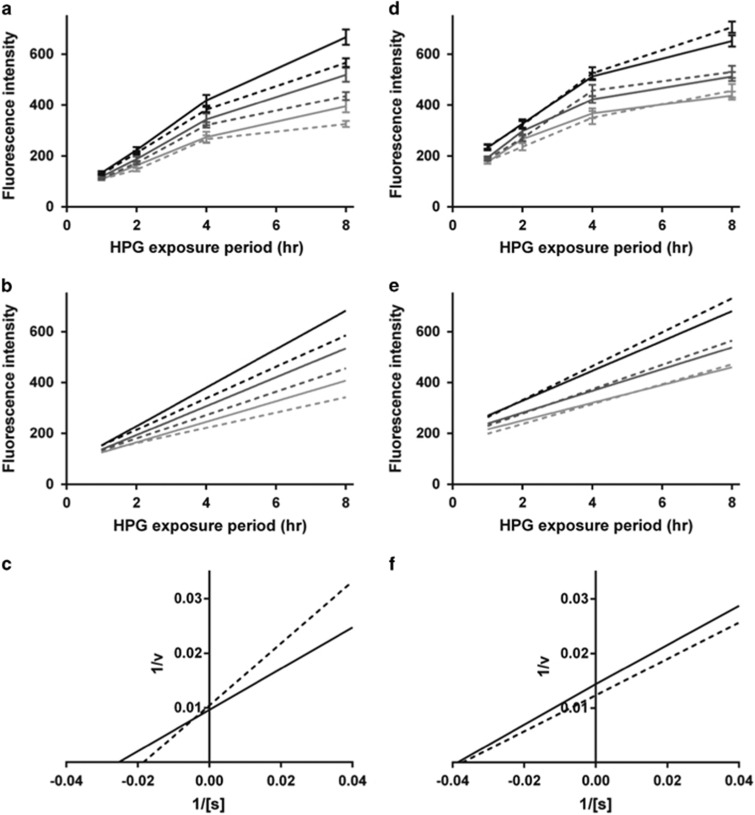
Reduced rates of global protein synthesis in schizophrenia patient-derived olfactory neurosphere-derived (ONS) cells in comparison with controls. Regression model illustrating global protein synthesis was reduced in nine patient-derived cell lines in comparison with nine control cell lines, but not in fibroblasts from the same patients. (**a**) L-homopropargylglycine (HPG) fluorescence at 1-, 2-, 4- and 8-h exposure periods in control-derived ONS cells (lines) and patient-derived ONS cells (dashed lines) at different HPG concentrations (100 μM, black; 50 μM, mid-gray; 25 μM, light gray). (**b**) Linear regression of the HPG fluorescence shown in **a**. (**c**) Lineweaver–Burk plots for the global protein synthesis reactions in control-derived ONS cells (line) and patient-derived ONS cells (dashed line) calculated from the slopes of the regression lines shown in **b**. The slopes of these lines are significantly different from each other (F_1,2_=79.98, *P*=0.012, *R*^2^=0.94). (**d**–**f**) Results for global protein synthesis rates in control-derived fibroblasts (line) and patient-derived fibroblasts (dashed line) from the same patients. The slopes of these lines were not significantly different from each other (F_1,2_=0.0707, *P*=0.815, *R*^2^=0.94).

To address the question of whether reduced ribosomal protein levels lead to reduced rates of protein synthesis in patient-derived ONS cells, we quantified global protein synthesis rates using a fluorescence incorporation assay for the methionine analog HPG. Experiments were performed on all nine schizophrenia patient-derived and nine control-derived ONS cell lines, at three HPG concentrations, over four time points. Images were captured at three wavelengths to detect nuclei (350 nm, 4,6-diamidino-2-phenylindole), cell cytoplasm (647 nm, CellMask) and newly synthetized protein (HPG, 488 nm; Figure 3a–c, respectively). HPG fluorescence was quantified as the mean intensity per pixel per cell, and quantification was based on analysis of 255 369 ONS cells across the nine patient-derived and nine control-derived cell lines. Although not obvious to the eye, schizophrenia-derived ONS cells were less fluorescent than control-derived ONS cells (Figure 3e–l), whereby ANOVA (Figure 4) found a significant effect for the disease status (F_1,396_=23.86, *P*<0.0001), confirming a schizophrenia-based effect on global protein synthesis, which was sustained for 8 h. There were also significant effects of HPG concentration (F_2,396_=112.62, *P*<0.0001) and exposure time (F_3,396_=649.68, *P*<0.0001), indicating that, as expected, all cells increased their fluorescence when exposed to a higher concentration of HPG and for longer periods.

Linear regression was used to derive the rates of protein synthesis at each HPG concentration (Figure 4b), and the goodness of fit for these regression lines was estimated from the correlation coefficient, R^2^, for each HPG concentration. The slopes of the regression lines were used in a linear regression to calculate Lineweaver–Burk plots (Figure 4c; R^2^=1.00), from which the rate constant (*K*m) and maximum reaction velocity (*V*max) were calculated. The *K*m was higher for patient-derived ONS (54 versus 40 μM), and the *V*max was lower (95 versus 104 μM h−1), in comparison with control-derived ONS cells, confirming that the control cells had a faster rate of protein synthesis (smaller *K*m) and larger capacity for protein synthesis (larger *V*max). ANOVA of the Lineweaver–Burk regressions was significant (F_1,2_=79.98, *P*=0.012), indicating that the slopes were significantly different.

We then asked whether the reduction in protein synthesis was cell-type-specific by quantifying protein synthesis rates in fibroblasts from the same individuals, and found that control and schizophrenia patient-derived fibroblasts were of similar fluorescence (Figure 4d–f). Furthermore, ANOVA demonstrated no significant effect of the disease status (F_1,312_=0.561, *P*=0.454). To address the question of whether reduced protein synthesis is correlated with reduced cell size, cell area was quantified using the cytoplasmic CellMask stain. This analysis found that schizophrenia patient-derived ONS cells were smaller than control-derived cells (2671+42 versus 3687+61 μm^2^; F_1,396_=184.75; *P*<0.0001). In contrast, schizophrenia patient-derived fibroblasts were larger than control-derived fibroblast cells (2650+95 versus 2151+29 μm^2^; F_1,336_=27.33; *P*<0.0001). Overall, these findings demonstrate that the reduced expression of ribosomal proteins in schizophrenia patient-derived ONS cells was associated with a significant reduction in the rate of protein synthesis compared with ONS cells from healthy controls.

### Upstream translational control pathways eIF2/4 and mTOR signaling are dysregulated in patient-derived ONS and iPSC models

As mRNA and protein expression levels are biochemically linked, we integrated our proteomic data with transcriptomic mRNA data from the same ONS cell lines using the Ingenuity Pathway Analysis tool.^10^ Our objective was to identify disrupted protein pathways, rather than single proteins; therefore, we uploaded the full list 102 significant proteins (*P*<0.05) for pathway analysis (Supplementary Data set 2). The most significant molecular pathways implicated (Benjamini–Hochberg FDR-adjusted Fisher's exact test at *P*<0.01) in both protein and mRNA data sets were eIF2 signaling, mTOR signaling and eIF4 signaling (Table 1; Supplementary Data set 3), all of which are upstream pathways concerned with the regulation of translational control and protein synthesis.^16^ No effects of smoking status and antipsychotic dose on protein expression were observed (data not shown). We next examined proteomic expression data from an independent study by Brennand *et al.*,^9^ asking whether the eIF2/4 and mTOR pathways were also dysregulated in schizophrenia patient-derived neuronal precursor cells generated from iPSCs. Pathway analysis of this proteomic data set confirmed that eIF2/4 and mTOR signaling were again the most significant pathways implicated (Benjamini–Hochberg FDR-adjusted Fisher's exact test at *P*<0.01; Supplementary Data set 4).

### Protein synthesis implicated in schizophrenia by genome-wide association studies

Finally, we reasoned that if the genes involved in eIF2 signaling are involved in the pathology of schizophrenia, then this may be reflected in population-based clinical studies. We tested this by performing gene-based association analysis,^17^ with molecules implicated in eIF2 signaling (corresponding to proteins and mRNA transcripts implicated in eIF2 signaling; Table 1) using genome-wide association data based on >13 000 schizophrenia cases and >18 000 controls.^14^ Two proteins showed a significant association with schizophrenia, *EIF2AK2* (*P*=3 × 10^−6^) and RPS13 (*P*=0.00029), following Bonferroni correction (*P*-value 0.05/48 genes tested=significance required *P*<0.0010; Supplementary Data set 5). *EIF2AK2* had the most significant gene-based score in the chromosome 2p22 region, whereas the neighboring gene to RPS13, *PIK3C2A*, is more significantly associated with schizophrenia (*P*=0.000126) than *RPS13*, thus not allowing us to verify the specific source of the *RPS13* association signal from this analysis (data not shown).

We then questioned whether all three biological pathways concerned with translational control—eIF2, mTOR and eIF4 signaling—were implicated in schizophrenia genomics data. We tested this by performing ‘Gene-set Enrichment Analysis'^15^ with molecules implicated in translational control (corresponding to the eIF2, eIF44 and mTOR proteins and mRNA transcripts in Table 1) in genome-wide association data,^14^ and the effect was significantly associated with schizophrenia following Bonferroni correction (*P*=0.019; Supplementary Data set 5), and showed a trend association with bipolar disorder (*P*=0.084), but not Crohn's disease or type 2 diabetes (Supplementary Data set 5).

## Discussion

We used a proteomics expression profiling approach to identify co-regulated protein pathways disrupted in schizophrenia patient-derived ONS cells in comparison with controls. Of the 102 proteins that were differentially expressed, 17 were ribosomal proteins that were significantly decreased, and several ribosomal/eIF2 signaling proteins (RPL14, RPL13A, RPL18A, PPP2R1A and FNRB) were robust discriminators between the control and schizophrenia groups (Figure 1). Led by these findings, we quantified global protein synthesis rates and demonstrated that patient-derived cell lines had significantly reduced rates of protein synthesis compared with control-derived cell lines. In addition, data integration analyses showed that the protein expression data converged with mRNA expression, and genome-wide association studies highlighted dysregulated eIF2α, mTOR and eIF4 signaling pathways, as correlating with the disease status.

Our findings of reduced cell area, ribosomal protein expression and rates of protein synthesis are consistent with previous post-mortem studies in schizophrenia, showing reduced neuronal somal size^18, 19^ and imaging studies that report reductions in whole-brain volume^20^ in schizophrenia. In keeping with the current study, we also recently reported altered ribosomal protein expression (RPLP1, RPLP2, RPL30 and RPL19) and Cytoplasmic FMR1 Interacting Protein 2 (CYFIP2) in post-mortem brain in schizophrenia.^21^ CYFIP regulates protein synthesis at the synapse by coordinating local mRNA translation and cytoskeleton remodeling to ensure proper dendritic spine formation.^22, 23, 24^ Previous stem cell studies of schizophrenia reported altered cellular migration and motility,^7, 9^ and our findings of altered expression of proteins involved in cytoskeletal remodeling (ARPC2, CYFIP2, GNAI2, GNB1, ITGB1, PDIA3, PFN2 and TLN1) and cellular adhesion and migration (ILK, ITGB1, PPP2CA, PPP2R1A, ITGB1, GMAI2, GNB1 and ARPC2) are consistent with these studies, and with previous post-mortem brain studies of schizophrenia.^25, 26^

The upstream signaling pathways concerned with translational control of protein synthesis are eIF2 and mTOR signaling,^16^ and we observed these pathways to be dysregulated in schizophrenia patient-derived ONS and iPSC models,^9^ and at the level of the genome in schizophrenia population-based data. In particular, eIF2 signaling is central to synaptic plasticity, learning and memory,^27^ and, given that genes involved in synaptic function are strongly implicated in schizophrenia risk,^28, 29^ it is reasonable to propose that dysregulation of protein synthesis via eIF2 signaling may contribute to schizophrenia. We tested the association of the 48 differentially expressed eIF2 pathway proteins and mRNA transcripts in a genome-wide association studies of >13 000 schizophrenia patients^14^ and found that15 eIF2 pathway proteins reached significance, and two (EIF2AK2 and RPS13) were highly significantly associated with the schizophrenia phenotype. In particular, EIF2AK phosphorylates eIF2α in response to viral infection, which in turn blocks the translation of viral mRNAs and protein synthesis.^30, 31^ This is particularly relevant, given the current literature implicating prenatal infection and inflammation as risk factors for schizophrenia.^32, 33^

The mTOR and eIF4 signaling pathways are also critical regulators of protein synthesis and ribosomal biogenesis, and have been previously implicated in neuropsychiatric disorders.^34^ Through phosphorylation of its downstream eIF4-binding proteins, mTOR complex 1 promotes protein synthesis,^34^ and direct pharmacological inhibition of mTORC1 blocks L-LTP and LTM.^35, 36^ Moreover, the antipsychotic medication haloperidol was recently shown to activate mTORC1 and enhance protein synthesis via eIF4 signaling in cultured neurons.^37^ In this context, it is important to note that regulation of protein synthesis may be cell-type-dependent. We observed deficits in protein synthesis rates in ONS cells but not in fibroblasts from the same patients, and these findings are supported by previous literature (for review see ref. 38). Given the potential disruption of all three translational control mechanisms eIF2, mTor and eIF4 signaling, which ultimately converge on altered protein synthesis, we performed gene-set enrichment analysis to test the association of these pathways in genome-wide association studies from individuals with schizophrenia and found a significant association with the schizophrenia phenotype.

In summary, our study has integrated data from proteomic, transcriptomic and *in vitro* functional assays in schizophrenia-patient-derived ONS cells, and genomic analyses, to provide important evidence that disturbed protein synthesis is associated with schizophrenia and could contribute to the development of the disorder. Future work is required to elucidate the specificity of these changes in the context of other neuropsychiatric disorders, and to determine the consequences of dysregulated protein synthesis at different developmental stages and in different cell types (for example, neurons versus glial cells during development). Overall, these data point to the dysregulation of protein synthesis in schizophrenia and suggest new molecular targets for intervention.

## Figures and Tables

**Table 1:** Pathway analysis of differentially expressed proteins and mRNA transcripts (Matigian *et al.*^10^) in patient-derived ONS cells in comparison with controls

*Rank*	*Significant pathways*	*IPA analysis*	*−Log (B-H P-value)*	*IPA ratio (number of Mol)*	*Number of implicated/total molecules*	*Molecules*
1	EIF2 signaling	Scz_ONS_mRNA	4.83E00	1.93E−01	39/202	MAPK1, PIK3R1, RPS18, RPL31, HRAS, RPL14, RPL26, RPS4Y1, RPL7, RPL10A, EIF2A, RPS11, RPS3A, RPS9, EIF5, RPL19, GSK3B, RPL8, RPL13, ATM, RPL4, EIF3H, RPL3, EIF3F, GRB2, RPS28, RPL12, EIF2B3, EIF3E, RPS27L, LOC100361644, FAU, RPS13, RPL28, AGO3, PIK3CD, EIF2AK2, RPL13A, RPS14
	EIF2 signaling	Scz_ONS_proteins	1.42E01	8.91E−02	18/202	RPL4, RPL27A, RPL3, RPL27, RPL17, RPS6, RPL14, RPL9,RPL10A, RPL6, RPS4X, RPL18A, EIF3D, RPL32, RPS3, RPL13A, RPL13, RPS14
2	mTOR signaling	Scz_ONS_mRNA	4E00	1.77E−01	37/209	MAPK1, PPP2R2A, PIK3R1, RPS18, RPS6KA3, FKBP1A, HRAS, RICTOR, RPS4Y1, RPS11, PGF, HMOX1, MTOR, RPS3A, RHOT1, RPS9, RPS6KA2, PRKD1, AKT1S1, EIF4B, PRKCA, ATM, MAPKAP1, EIF3H, EIF3F, RHOC, RPS28, RHOJ, EIF3E, RPS27L, FAU, RPS13, PPP2R3A, PPP2R2B, PRR5, PIK3CD, RPS14
	mTOR signaling	Scz_ONS_proteins	2.09E00	3.35E−02	7/209	RPS4X, PPP2R1A, PPP2CA, EIF3D, —, RPS3, RPS14
3	Regulation of eIF4 signaling	Scz_ONS_mRNA	2.73E00	1.61E−01	28/174	MAPK1, PPP2R2A, PIK3R1, RPS18, HRAS, RPS4Y1, EIF2A, RPS11, MTOR, RPS3A, EIF4EBP3, RPS9, ATM, EIF3H, EIF3F, GRB2, RPS28, EIF2B3, EIF3E, ITGA3, RPS27L, FAU, RPS13, PPP2R3A, PPP2R2B, AGO3, PIK3CD, RPS14
	Regulation of eIF4 signaling	Scz_ONS_proteins	3.62E00	4.6E−02	8/174	ITGB1, RPS4X, PPP2R1A, PPP2CA, EIF3D, —, RPS3, RPS14
4	Ephrin receptor signaling	Scz_ONS_mRNA	2.73E00	1.56E−01	31/199	FYN, MAPK1, PTPN13, GNA11, HRAS, GNG13, MAP4K4, CRK, NCK1, PGF, ROCK2, NCK2, EPHB6, PAK1, ARPC4, EFNB3, SRC, PAK4, CFL1, GRB2, ARPC5L, GNAI1, ITGA3, RAC3, ATF2, WIPF1, ABI1, ARPC2, ACP1, PDGFD, EPHA2
	Ephrin receptor signaling	Scz_ONS_proteins	8.6E−01	2.01E−02	4/199	ITGB1, GNAI2, GNB1, ARPC2
5	ILK signaling	Scz_ONS_mRNA	2.57E00	1.67E−01	32/192	MAPK1, PPP2R2A, PIK3R1, RICTOR, PPP1R14B, CCND1, PGF, NCK2, PARVB, MTOR, RHOT1, ILKAP, GSK3B, ITGB4, ATM, NACA, CFL1, TMSB10/TMSB4X, RHOC, TNFRSF1A, ACTB, FERMT2, MAPK9, RHOJ, MYL6B, ATF2, FOS, DOCK1, PPP2R3A, SNAI2, PPP2R2B, PIK3CD
	ILK signaling	Scz_ONS_proteins	8.39E−01	2.08E−02	4/192	ITGB1, PPP2R1A, PPP2CA, ILK

Abbreviations: IPA, ingenuity pathway analysis; ONS, olfactory neurosphere-derived.

The top five pathways are listed in order of significance (*P*-value), which was determined using the right tailed Fisher exact test in IPA, and by the IPA ratio. The table details the significant ONS proteins and mRNA transcripts that map to eIF2, mTOR, eIF4, Ephrin and ILK signaling pathways.

## Supplementary Material

Supplementary Table 1

Supplementary Datasets
