# Supplementary material for: Reduced protein synthesis in schizophrenia patient-derived olfactory cells
Source: Transl Psychiatry. 2015 Oct 20;5(10):e663–. doi: 10.1038/tp.2015.119 (PMC4930119; doi:10.1038/tp.2015.119)

Supplementary Figure 1 (a) Example of targeted MS1 analysis in Skyline. The peak area for triply changed peptide SYCAEIAHNVSSK, from protein RPL32, with a mass of 489.2295, and a retention time of 18 – 19min, is illustrated for the control (top three peaks) and schizophrenia (lower three peaks), in technical replicates of pooled data (N = 9 per diagnostic group).

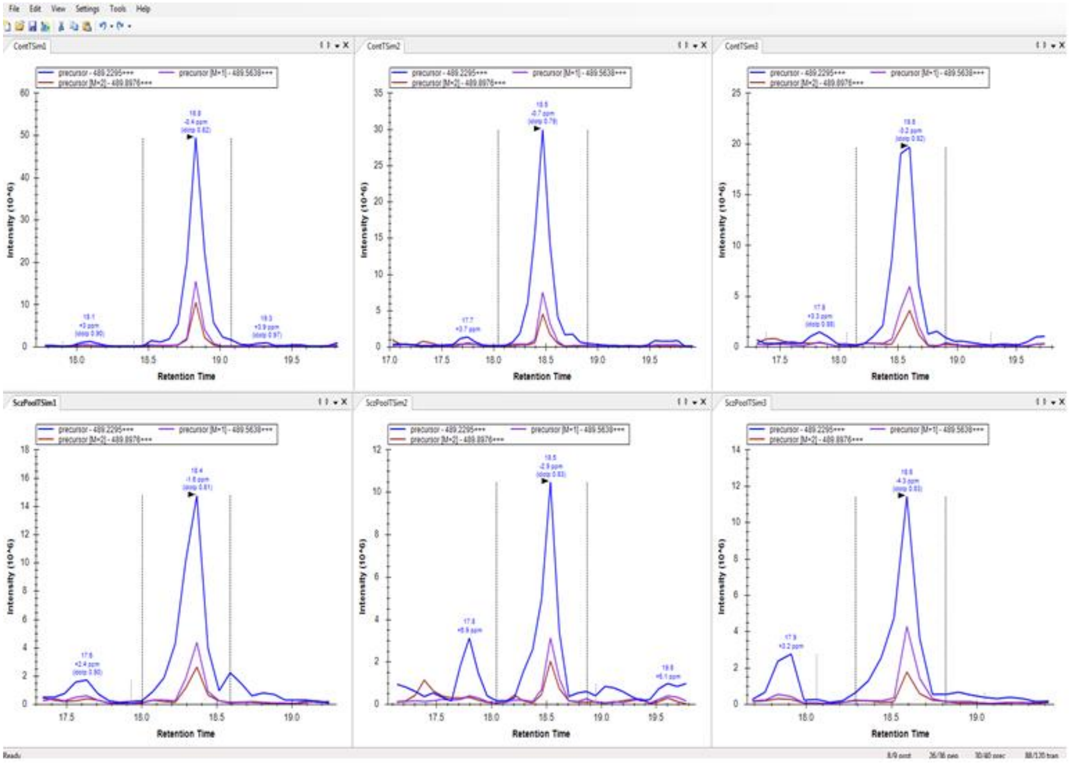

(b) Western blotting confirmed significant decreases in eIF2α, RPL13A, and RPL18A (p<0.05) in schizophrenia (S) derived ONS stem cells in comparison to controls (C), thus validating our proteomic findings. Significance is indicated by p<0.05*, p <0.01**, p<0.001***.

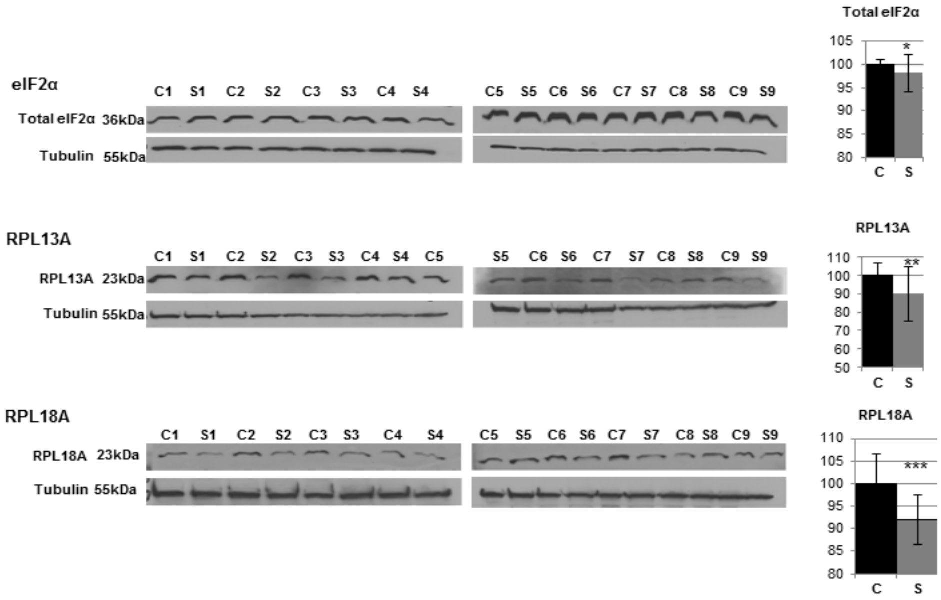

Supplement: Supplementary Figure 1 [file tp2015119x1.pdf]
