# Supplementary material for: Reduced protein synthesis in schizophrenia patient-derived olfactory cells
Source: Transl Psychiatry. 2015 Oct 20;5(10):e663–. doi: 10.1038/tp.2015.119 (PMC4930119; doi:10.1038/tp.2015.119)
Supplement: Supplementary Table 1 [file tp2015119x2.pdf]

Supplementary Table 1: Patient demographics.

The table list the human olfactory neurosphere-derived cell lines used in this study, including disease status, gender, age, smoking status (Y, yes ; N, no), and medications taken at the time of biopsy. For comparative purposes, medication d doses are converted to chlorpromazine (CPZD) equivalents.

Disease Status	Gender	Age	Smoking Status	Medication	Dose (Chlorpro mazine	Other Medication	Passage No.
Control	Male	31	Y				8
Control	Male	47	N				7
Control	Male	28	N				5
Control	Male	17	N			Salbutamol, Fluticas	11
Control	Male	24	N				7
Control	Male	32	N				6
Control	Male	46	N				5
Control	Male	56	N				8
Control	Male	45	Y				7
Schizophrenia	Male	46	Y	Clozapine	333	Omeprazole magnes	5
Schizophrenia	Male	58	N	Olanzapine	250	Benztropine, Diclofe	6
Schizophrenia	Male	21	Y	Quetiapine	1194	Paroxetine	9
Schizophrenia	Male	33	N	Risperidone	267		5
Schizophrenia	Male	49	Y	Colzapine	467		8
Schizophrenia	Male	27	N	Olanzapine	533		11
Schizophrenia	Male	44	Y	Clozapine	633	Lithium carbonate, /	7
Schizophrenia	Male	28	Y	Flupenthixol de	500		8
Schizophrenia	Male	38	N	Risperidone	unknown		5
