# Supplementary material for: Reduced protein synthesis in schizophrenia patient-derived olfactory cells
Source: Transl Psychiatry. 2015 Oct 20;5(10):e663–. doi: 10.1038/tp.2015.119 (PMC4930119; doi:10.1038/tp.2015.119)
Supplement: Supplementary Material [file tp2015119x4.doc]

**SUPPLEMENTARY MATERIAL**

**___________________________________________________________________________**

**EXTENDED EXPERIMENTAL PROCEDURES**

***Human Olfactory Neurosphere-Derived (ONS) Cells***

Olfactory mucosa biopsies were obtained from 9 male schizophrenia and 9 male controls, and grown as neurospheres, as previously described . Briefly, ONS cells are derived from neurospheres that form when dissociated olfactory mucosal cells are grown in a serum-free medium containing epidermal growth factor (EGF) and fibroblast growth factor 2 (FGF2). Neurospheres were generated from all patients and controls, with no obvious disease-related differences in neurosphere growth and formation. The neurospheres were dissociated and grown in the presence of serum as an adherent monolayer of ONS cells. We checked gene expression stability in one cell line from passages 7-25. At passages 10, 13, 16, 19, 22 and 25 the gene expression profile (across the 10,000 expressed genes) was correlated with the gene expression profile at passage 7, yielding correlation coefficients of 0.99, 0.98, 0.98, 0.96, 0.94 and 0.94, respectively. This high level of correlation indicates that ONS cells are quite stable genetically for many passages and more than 100 days in vitro[^1^](#_ENREF_1).

Case-control status was determined with the Diagnostic Interview for Psychosis according to criteria from the DSM-IV (American Psychiatric Association, 1994). For details on patient demographics, please refer to Supplementary Table 1. Procedures were carried out in accordance with the ethics committees of The Park Centre for Mental Health and Griffith University, according to guidelines of the National Health and Medical Research Council of Australia. Frozen aliquots of cells were thawed and grown as described previously [^1^](#_ENREF_1). All assays were done with cells cultured for similar periods after nasal biopsy (between 5 and 11 passages from the initial plating). The average passage numbers were the same for patient-derived ONS cells and control-derived ONS cells at the time of protein extraction (patient-derived: 7+2.1, n=9; control-derived: 7+1.8, n=9). The ONS cells derived from schizophrenia and control subjects have similar morphologies, with similar proportions of cells expressing the different antigens representative of markers for bone marrow stromal cells, neural stem cells, neurons, and glia as previously described [^1^](#_ENREF_1) .

***Proteomic analysis of ONS Cells by Label Free LC-MS/MS***

Prior to label free LC-MS/MS analysis, ONS cells were washed twice with 10ml of HBSS and harvested using a cell scraper to detach cells. The cells were pelleted by centrifugation at 400 x g for 5 min and the supernatant was discarded. The HBSS were washed again to ensure removal of serum, and the pellet was resuspended in 2ml of Triethylammonium bicarbonate (TEAB; Sigma). Following solubilisation of samples by sonication in triethylammonium bicarbonate (TEAB; Sigma), the protein concentration was determined using a Bradford Assay[^5^](#_ENREF_5), according to the manufacturer (BioRad) instructions. Note that because there was significantly less protein (p=0.018; data not shown) in the schizophrenia ONS cells (1.28µg/µl), in comparison to control ONS cells (1.61µg/µl), samples were normalised by protein concentration, whereby equal concentrations of protein (1μg per sample) were injected on the mass spectrometer for relative comparison of protein expression profiles between the groups.

Proteomic analysis by label free LC-MS/MS analysis was carried out as previously described [^6^](#_ENREF_6), whereby 1µg of protein for each of the 18 samples was injected, in triplicate, on a Thermo Scientific LTQ ORBITRAP XL mass spectrometer. Survey full-scan MS spectra (300–2000 Da) were acquired in the Orbitrap with a resolution of 60,000 and the 7 most intense ions from the parent ion scan were selected for MS/MS analysis [^6^](#_ENREF_6). Label-free quantification (LFQ) was performed with Max Quant (V1.3.0.2) as described . Protein and peptide FDR’s were set to 0.01, and only proteins with at least two peptides (one uniquely assignable to the protein) were considered as reliably identified. LFQ intensity values were used for protein quantification between groups. Statistical analysis was performed in Perseus (V 1.3.0.4), whereby the data was log2 transformed to eliminate distributional skew and improve the normal approximation for validity of p-values. Data imputation was used to replace Missing values by values form the normal distribution, and data normalisation was performed by subtracting the median LFQ intensity per case (Figure 1a). Student’s t-test was then applied to identify proteins differentially expressed between groups at a 5% threshold, and a permutation-based FDR was applied at a 10% threshold (Supplementary Table 2). To rule out smoking status as a potential confound of protein expression results, a second analysis was performed, as above, on non-smokers from the control (n = 7) and schizophrenia (n = 4) diagnostic groups, and the students t-test was used to identify differential protein expression between non-smoking groups. Please refer to Supplementary Table 1 for detailed patient demographics. Likewise, to rule out medication effects the schizophrenia subjects were divided into low (n = 3) and high (n = 5) dose Chlorpromazine Equivalents (CPZE), and analysis was performed as above, whereby the Students t-test was used to identify proteins differentially expressed between low and high dose CPZE schizophrenia patients.

***Confirmation of Protein Changes by Targeted Mass Spectrometry***

For confirmation of protein changes by targeted MS, schizophrenia and control samples were pooled according to diagnosis, and samples spiked with Pierce Retention Time (RT) Calibration Mixture, a mixture of 15 heavy isotope-labelled reference peptides, according to the manufacturer’s instructions (Thermo Scientific Product No. 88321). Target peptides were selected from Max Quant data acquired in the label-free LC-MS/MS study as described above.

For unscheduled RT prediction [^8^](#_ENREF_8), samples (1ug) were injected on the Thermo Q-Exactive equipped with a Dionex Ultimate 3000 (RSLCnano) chromatography system, and separated onto Biobasic Picotip Emitter (120 mm length, 75 μm ID) packed with Reprocil Pur C18 (1.9 μm) reverse phase media, and was separated using a 60 min reverse phase gradient at a flow rate of 250 nL/min. Survey full-scan MS spectra (300–1600 Da) were acquired in the Q-Exactive with a resolution of 140,000, whereby the 12 most intense ions from the preview scan were selected for HCD. For the targeted analysis by single ion monitoring (SIM) an isolation list detailing the mass and RT for target peptides was uploaded to Thermo XCalibur (V2.2 SP1.48), and each sample (1ug) was run in triplicate, on the Thermo Q-Exactive, and chromatographically separated as described above. A targeted SIM (tSIM) scan was acquired at a resolution of 35,000, from 300 to 1600 *m/z*, for target peptides listed on the isolation list (Supplementary Table 5), where the RT window for each target peptide was set to 4 min.

Bioinformatics software, Skyline (v2.1.0.4936; [https://skyline.gs.washington.edu](https://skyline.gs.washington.edu/)) was used for MS1 filtering [^9^](#_ENREF_9) and RT prediction [^8^](#_ENREF_8) of target peptides. Briefly, a spectral library was created from the raw MS/MS data generated in the unscheduled run. These files were also used for initial RT prediction and tuning the data for MS1 filtering. An isolation list, with a RT window of 4 min for each target peptide, was exported from Skyline for targeting with a scheduled run on the Q-Exactive. For targeting, tSIM files were imported into a new Skyline document, along with the peptide targets for each candidate protein, to enable MS1 filtering and quantitation of selected precursor ions. For these High Resolution/Accurate Mass (HR/AM) scans of isolated peptides, Skyline enabled confident peak picking of multiple precursors, using the isotopic disruption (idotp), mass accuracy, and RT properties for each target peptide, across replicate runs (Supplementary Figure 1a). Precursor data was exported from Skyline and the relative peptide abundance was measured (i.e. “area under the curve”) for proteins of interest, taking the median abundances of peptides per protein [^10^](#_ENREF_10).

***Confirmation of Protein Changes by Western blotting***

Confirmation of protein changes by western blotting was undertaken on four proteins eIF2, phospho-eIF2, RPL13A and RPL18A, in schizophrenia and control ONS cells, whereby each of the 18 samples were run in triplicate. We selected proteins for validation based on their degree of differential expression, the rank of the fold change differences in expression, availability of antibodies, and pathway analysis results. Equal concentrations of denatured protein homogenates (5-20 μg/lane, dependent on the antibody) were loaded and resolved on 12% SDS-polyacrylamide gels, the proteins were separated by electrophoresis and transferred onto nitrocellulose membranes. Each protein migrated with a single band at the predicted molecular weight, elF2α (36 kDa, Invitrogen, 1:2000), elF2α [pS^52^] (36 kDa, Invitrogen, 1:2000), RPL13A (23 kDa, Cell Signalling Technology, 1:1000), and RPL18A (23 kDa, Proteintech, 1:1000) were used as primary antibodies. The corresponding horseradish peroxidase-conjugated secondary antibodies (anti-rabbit IgG (1:2000-1:4000, Promega) and anti-mouse IgG (1:2000, Promega)) were used. Blots were incubated with ECL chemiluminescent reagent (Amersham Biosciences), exposed to X-ray film and developed accordingly. Levels of expression were quantified and normalized against anti-(mouse) Tubulin βIII (1:5000, Upstate/Millipore). Density values were measured (Adobe Photoshop) and corrected by the signal intensity of the respective antibody to Tubulin βIII. Band densities were normalized against Tubulin βIII since tubulin proteins were found to be unchanged across cases and controls in our label free proteomics data (Supplementary Table 2). Statistical significance was set at the 5% level (Supplementary Figure 1b).

***Protein Translation Assay in ONS cells using Click Chemistry for HPG***

Global protein synthesis was quantified in schizophrenia and control ONS cells using copper catalyzed azide-alkyne cycloaddition (“Click Chemistry”). ONS cells and fibroblasts were grown in Dulbecco’s minimum essential medium (DMEM, Gibco) with F12 supplement (Gibco) supplemented with 10% fetal bovine serum. When the cell culture reached 80% confluence the cells were released from the culture flask and dissociated into a single cell suspension (using TrypeLE, Gibco). 3000 cells (100 μl) were seeded into fibronectin- coated (10 μg/ml) wells of 96-well plates and allowed to grow for 24 hr. The cells were washed once with warm phosphate buffered saline (PBS, pH7.4) and cultured in DMEM without L-Methionine for 1 h then in DMEM containing 25, 50, or 100 μM HPG (L-homopropargylglycine) and cultured for 1h, 2h, 4h or 8h at 37°C and 5% CO_2_ for 1h, 2h, 4h and 8h. Each plate contained cells from all patient-derived and control-derived cell lines exposed to 3 HPG concentrations for one of the time periods, requiring 4 plates to cover all time periods. This experiment was duplicated with an additional 4 plates. The same experiment design was used for ONS cells and for fibroblasts. The HPG was diluted from 100 mM stock solution in dimethyl sulfoxide (DMSO). Medium containing DMSO was used as negative control.

HPG content was detected according to the manufacturer’s protocol (“Click-IT”, Invitrogen). Cells were fixed in 4% paraformaldehyde in PBS for 15 min, permeabilized with 0.25% Triton X-100 in PBS for 15min, washed once with 3% bovine serum albumin (BSA) in PBS, and incubated in 100 μl Click-IT reaction cocktail (1x Click-IT reaction buffer and buffer additive, 2mM CuSO_4_, 3 μM Alexa Fluor 488-Azide) added to each well for 30 minutes at room temperature in the dark, followed by a wash with 3% BSA in PBS. The cell cytoplasm was stained by 1x CellMask staining solution (1:10,000, Invitrogen) for 30 min at room temperature and nuclei were stained by DAPI (1:1000, Invitrogen) in PBS for 10 minutes, followed by a wash with PBS.

Images were analysed by automated microscopy (Operetta High Content Imaging System, Perkin Elmer). Images were captured at 56 locations per well at 200× magnification at three wavelengths (488nm, HPG; 647nm, CellMask; 350nm, DAPI). The three images were combined and analyzed using Harmony High Content Analysis Software (Perkin Elmer). The analysis protocol involved the following steps: 1) each cell nucleus was identified using the DAPI stain, 2) the cell cytoplasm was defined from CellMask fluorescence, 3) cells that overlapped the border of the image frame were excluded from the analysis, 4) newly synthesised protein fluorescence intensity was quantified within each cell cytoplasm defined as the region between the nucleus (DAPI-labelled) and the outer edge of the cell (CellMask-labelled). All images were taken with the same time and exposure settings. Each analysis included cells from the 9 schizophrenia and 9 control cases, at 3 different HPG concentrations, in the same 96-well plate. Duplicate plates were used for each time-point. To eliminate observer bias, the image analysis was automated using the same parameters for every image. This analysis provided mean HPG fluorescence per pixel per cell for each schizophrenia- and control-derived cell line.ONS cells and fibroblasts were grown, analysed and quantified in separate experiments. To address the question of whether reduced levels of ribosomal proteins and global protein synthesis were associated with changes in cell size we also quantified cell area defined by the cytoplasmic CellMask stain.

The final analysis is based on a total of 255,369 ONS cells selected according to the automated analysis, based on 9 schizophrenia-derived and 9 control-derived ONS cell lines. The average number of cells analysed per HPG concentration per time period was similar for the schizophrenia-derived and control-derived groups (schizophrenia: 598+37.9; control: 614+22.8). Similar numbers of fibroblasts were analysed: total number of fibroblasts, 243,768; average numbers of fibroblasts per HPG per time period: schizophrenia: 695+15.5; control: 756+37.4). All data are expressed as mean + SEM. Differences in protein synthesis between schizophrenia- and control-derived ONS cells and fibroblasts were assessed in a three-way ANOVA with Status (Control, Schizophrenia), HPG Concentration (25, 50, or 100 μM) and Exposure Time (1-8 hr) as Main Effects (IBM SPSS v21). Alpha was set to 0.05. The slopes of the reaction curves were calculated for each concentration of HPG using linear regression (GraphPad Prizm). These slopes and concentrations were used to generate Lineweaver-Burk plots from which Km and Vmax were calculated using linear regression (GraphPad Prizm).

***Ingenuity Pathway Analysis of Proteomic and mRNA Data from ONS Cells***

Ingenuity Pathway Analysis (IPA; [www.ingenuity.com](http://www.ingenuity.com/)) was performed on all 102 statistically significant proteins identified in our label free proteomic study (Supplementary Table 2). In addition, statistically significant mRNA expression data from the same samples[^1^](#_ENREF_1) was imported and a “comparative analysis” was undertaken in IPA. The IPA p-value was calculated using the right-tailed Fisher’s Exact Test and multiple hypothesis correction was based on the Benjamini-Hochberg approach at 1% FDR threshold (Table 1; Supplementary Table 3). A second pathway analysis was undertaken on proteomic data recently published by Brennand and colleagues[^11^](#_ENREF_11) in order to investigate the top protein pathways disrupted in schizophrenia-derived Neuronal Progenitor Cells (NPC’s) that were differentiated from human induced Pluripotent Stem Cells (hiPSC). The proteomic data from Brennand and colleagues (2014)(Supplementary Tables 5-8) comprised of four independent Patient vs Control hiPSC NPC’s quantitative SILAC MS analyses. We imported proteins deemed statistically significant[^11^](#_ENREF_11) (Supplementary Tables 5-8; ANOVA p-value with BH correction < 0.05)[^11^](#_ENREF_11) for the four hiPSC NPC’s MS analyses into IPA. All 4 datasets (P1A vs C1_hiPSC NPC’s; P3 vs C3_hiPSC NPC’s; P3 vs C3_hiPSC NPC’s; and P3 vs C6 hiPSC NPC’s) were processed together via IPA analysis. The IPA p-value was calculated using the right-tailed Fisher’s Exact Test and multiple hypothesis correction was based on the Benjamini-Hochberg (B-H) approach at 1% for FDR threshold. Results for this analysis are presented in Supplementary Table 4.

***Testing for associations with Schizophrenia in Genome-Wide Association Data***

Single gene based analysis was tested on the differentially expressed genes and proteins in the eIF2 signalling pathway (Table 1). Genome wide SNP association results were available from the Psychiatric Genetic Consortium on a meta-analysis on two samples, the Swedish National Sample (5,001 cases and 6,243 controls), and a previous PGC study (8,832 cases and 12,067 controls) on 9,898,078 SNPs imputed to 1000G [^12^](#_ENREF_12). A gene-based test was performed using VEGAS software which summarized the evidence for SNP association with schizophrenia on a per-gene basis (including SNPs +/- 50kb outside of genes to include regulatory regions) by considering the p-values of all SNPs (n=9,898,078) within 17,769 unique autosomal genes [^13^](#_ENREF_13). First, genes that mapped to the differentially expressed molecules in the eIF2 signalling pathways were tested for association with schizophrenia (n=48 genes), two genes were unable to be tested as RPS4X is on the x Xchromosome, and RPS4Y1 is on the Y chromosome. Individual candidate genes were considered significant, if they surpassed a Bonferroni level of correction (P 0.05/total number of genes tested = 48 genes, p≤ 0.0010). Regional association plots were created used LocusZoom (Pruim et al, 2010) and results are presented in Supplementary Table 6a.

In a second analysis, gene-sets of EIF2 signalling (n=48 genes), mTOR signalling (n=40 genes), and EIF4 signalling (n=33 genes) were tested for enrichment with schizophrenia. The enrichment of the gene-sets was tested using a competitive test of enrichment, GSEA v2.0 . The gene-based p-values of association with schizophrenia were –log (10) transformed and rank ordered. GSEA tested the ranking of the genes in our gene-sets within the genome-wide set of gene association with schizophrenia using weighted enrichment statistics with 5,000 permutations, repeating the analysis three times. Gene-sets meeting the recommended discovery criteria of an uncorrected p-value <0.05, and FDR-corrected q-value <0.25 were considered significant. Results are presented in Supplementary Table 6b.

**REFERENCES**

1. Matigian N, Abrahamsen G, Sutharsan R, Cook AL, Vitale AM, Nouwens A*, et al*. Disease-specific, neurosphere-derived cells as models for brain disorders. *Dis Model Mech* 2010; **3**(11-12)**:** 785-798.

2. Fan Y, Abrahamsen G, McGrath JJ, Mackay-Sim A. Altered cell cycle dynamics in schizophrenia. *Biological psychiatry* 2012; **71**(2)**:** 129-135.

3. Fan Y, Abrahamsen G, Mills R, Calderon CC, Tee JY, Leyton L*, et al*. Focal adhesion dynamics are altered in schizophrenia. *Biological psychiatry* 2013; **74**(6)**:** 418-426.

4. Mackay-Sim A. Concise review: Patient-derived olfactory stem cells: new models for brain diseases. *Stem Cells* 2012; **30**(11)**:** 2361-2365.

5. Bradford MM. A rapid and sensitive method for the quantitation of microgram quantities of protein utilizing the principle of protein-dye binding. *Analytical biochemistry* 1976; **72:** 248-254.

6. English JA HA, Föcking M, Wynne K, Scaife C, Cagney G, Moriguchi T, Cotter DR. Omega-3 fatty acid deficiency disrupts endocytosis, neuritogenesis, and mitochondrial protein pathways in the mouse hippocampus *Frontiers Genetics* 2013; **10.3389/fgene.2013.00208**

7. Hubner NC, Bird AW, Cox J, Splettstoesser B, Bandilla P, Poser I*, et al*. Quantitative proteomics combined with BAC TransgeneOmics reveals in vivo protein interactions. *J Cell Biol* 2010; **189**(4)**:** 739-754.

8. Escher C, Reiter L, MacLean B, Ossola R, Herzog F, Chilton J*, et al*. Using iRT, a normalized retention time for more targeted measurement of peptides. *Proteomics* 2012; **12**(8)**:** 1111-1121.

9. Schilling B, Rardin MJ, MacLean BX, Zawadzka AM, Frewen BE, Cusack MP*, et al*. Platform-independent and label-free quantitation of proteomic data using MS1 extracted ion chromatograms in skyline: application to protein acetylation and phosphorylation. *Mol Cell Proteomics* 2012; **11**(5)**:** 202-214.

10. Cox J, Neuhauser N, Michalski A, Scheltema RA, Olsen JV, Mann M. Andromeda: a peptide search engine integrated into the MaxQuant environment. *J Proteome Res* 2011; **10**(4)**:** 1794-1805.

11. Brennand K, Savas JN, Kim Y, Tran N, Simone A, Hashimoto-Torii K*, et al*. Phenotypic differences in hiPSC NPCs derived from patients with schizophrenia. *Mol Psychiatry* 2014.

12. Ripke S, O'Dushlaine C, Chambert K, Moran JL, Kahler AK, Akterin S*, et al*. Genome-wide association analysis identifies 13 new risk loci for schizophrenia. *Nat Genet* 2013.

13. Liu JZ, McRae AF, Nyholt DR, Medland SE, Wray NR, Brown KM*, et al*. A versatile gene-based test for genome-wide association studies. *Am J Hum Genet* 2010; **87**(1)**:** 139-145.

14. Subramanian A, Tamayo P, Mootha VK, Mukherjee S, Ebert BL, Gillette MA*, et al*. Gene set enrichment analysis: a knowledge-based approach for interpreting genome-wide expression profiles. *Proc Natl Acad Sci U S A* 2005; **102**(43)**:** 15545-15550.

15. Wang K, Li M, Hakonarson H. Analysing biological pathways in genome-wide association studies. *Nature reviews Genetics* 2010; **11**(12)**:** 843-854.

16. Psychiatric GCBDWG. Large-scale genome-wide association analysis of bipolar disorder identifies a new susceptibility locus near ODZ4. *Nat Genet* 2011; **43**(10)**:** 977-983.

17. Morris AP, Voight BF, Teslovich TM, Ferreira T, Segre AV, Steinthorsdottir V*, et al*. Large-scale association analysis provides insights into the genetic architecture and pathophysiology of type 2 diabetes. *Nat Genet* 2012; **44**(9)**:** 981-990.

18. Anderson CA, Boucher G, Lees CW, Franke A, D'Amato M, Taylor KD*, et al*. Meta-analysis identifies 29 additional ulcerative colitis risk loci, increasing the number of confirmed associations to 47. *Nat Genet* 2011; **43**(3)**:** 246-252.

**SI: Figure, Table, and Dataset Legends**

**___________________________________________________________________________**

**Supplementary Figure Legends**

**SI Figure 1 (a)** Example of targeted MS1 analysis in Skyline. The peak area for triply changed peptide SYCAEIAHNVSSK, from protein RPL32, with a mass of 489.2295, and a retention time of 18 – 19min, is illustrated for the control (top three peaks) and schizophrenia (lower three peaks), in technical replicates of pooled data (N = 9 per diagnostic group). **(b)** Western blotting confirmed significant decreases in pEIF2α, EIF2α, RPL13A, and RPL18A (p<0.05) in schizophrenia (S) derived ONS cells in comparison to controls (C), thus validating our proteomic findings. Significance is indicated by p<0.05*, p <0.01**, p<0.001***.

**Supplementary Table Legends:**

**SI Table 1:** Details on patient demographics. The table list the human olfactory neurosphere-derived cell lines used in this study, including disease status, gender, age, smoking status (Y, yes ; N, no), and medications taken at the time of biopsy. For comparative purposes, medication doses are converted to chlorpromazine (CPZD) equivalents.

**Supplementary Datasets - Legends:**

**SI Dataset 1:** Results of the label-free LC-MS/MS proteomic analysis, in which 102 proteins were significantly differentially expressed (p<0.05) between control and schizophrenia-derived ONS cells. Of these 102 proteins, 56 were increased and 46 were decreased in expression in schizophrenia derived ONS cells. The table lists the gene symbol, proteins that were significant following FDR correction at 5% and 10%, as well as the t-test p value, t-test difference (fold change), protein ID, and LFQ intensity for each of the proteins identified in the 18 cell lines. Please also refer to Figure 1a, which illustrates the line plots of all proteins identified across the 18 samples, and overlaying boxplots illustrating the spread and median of the data for each case in the cohort.

**SI Dataset 2:** Extended Pathway analysis of significant proteins and mRNA transcripts (Matigian et al. 2010) in schizophrenia-derived ONS cells. All pathways are listed in order of significance (p-value) which was determined using the right tailed Fisher exact test in IPA, and by the IPA ratio, which is number of molecules in a given pathway that meet significance, divided by the total number of molecules that make up that pathway. The table details the significant ONS proteins and mRNA transcripts that map to each pathway tested.

**SI Dataset 3:** Extended pathway analysis used to identify the top signalling pathways implicated in schizophrenia-derived Neuronal Progenitor Stem Cell (NPC’s) differentiated from human induced Pluripotent Stem Cells (hiPSC’s)[^11^](#_ENREF_11). Our pathway analysis of quantitative mass spectrometry data from four independent Patient (P) vs Control (C) SILAC analysis (Brennand et al. 2014-Supplementary Tables 5-8) revealed that eIF2, eIF4, and mTOR were the top 3 scoring pathways implicated in this independent stem cell model and cohort of patients. Thus, the top-ranked cellular pathways found to differ in two cellular models of schizophrenia centres on translation control mechanisms.

**SI Dataset 4:** Isolation list exported from the Skyline software for targeted analysis with a tSIM scan on the Q-Exactive instrument, whereby the Retention Time (RT) was set to 4 minutes, as indicated by the Start (min) and End (min) columns for each peptide mass (m/z). The change state and polarity is also given for each peptide mass.

**SI Dataset 5.** Genomic analyses. **(a)** eIF2 signalling proteins (n=18) and mRNA transcripts (n=39; Matigian et al., 2010) that were significantly differentially expressed (p<0.05) in schizophrenia-derived ONS cells, in comparison to controls. The table lists the fold change (FC) observed at the protein, and mRNA level, as well indicates which proteins were confirmed as significantly decreased by western blotting (WB) or by targeted MS (tMS). The table also details the gene-based associations of the eIF2 pathway molecules, along with the chromosomal location, and most significant SNP for each region tested. Two of the eIF2 molecules were highly significantly association with schizophrenia; EIF2AK2 (P=3x10^-6^) and RPS13 (P=0.00029) following Bonferroni correction (P-value 0.05/48 genes tested = significance required P<0.0010).

**(b)** Results from the gene-set enrichment analysis, in which differentially expressed molecules that mapped to the eIF2, mTOR, and eIF4 signalling pathways were tested for association with schizophrenia. The analysis was performed comparably large GWAS studies of schizophrenia ([^12^](#_ENREF_12), bipolar disorder [^16^](#_ENREF_16), type 2 diabetes [^17^](#_ENREF_17), and Cohn’s disease [^18^](#_ENREF_18) ) to confirm that enrichment was specific to psychiatric illness.
